# Longitudinal assessment of the retinal phenotype in patients with Fabry disease undergoing continuous enzyme replacement and/or chaperone therapy

**DOI:** 10.1186/s13023-026-04654-2

**Published:** 2026-10-08

**Authors:** Annabelle Volk, Jan Wildner, Johannes Birtel, Simon Dulz, Luca Mautone, Anja Köhn, Nicole Muschol, Yevgeniya Atiskova

**Affiliations:** 1 Department of Ophthalmology, University Medical Center Hamburg-Eppendorf, Hamburg, Germany; 2 International Center for Lysosomal Disorders (ICLD), Department of Pediatrics, University Medical Center Hamburg-Eppendorf, Hamburg, Germany; 3 Eyelid Aesthetics, Neuer Wall 10, Hamburg, 20354 Germany

**Keywords:** Fabry disease, OCT, Retina, Hyperreflective foci, Vessel tortuosity, Morbus Fabry

## Abstract

**Purpose:**

Fabry disease (FD) is an X-linked inherited lysosomal storage disorder caused by deficiency of the enzyme α-galactosidase A. This study describes the long-term evolution of retinal findings in genetically confirmed FD patients, who have been receiving enzyme replacement therapy (ERT) or pharmacological chaperone therapy (PCT).

**Methods:**

This retrospective observational study included 42 eyes of 21 patients, who underwent comprehensive ophthalmic examination including best-corrected visual acuity (BCVA) testing, tonometry, slit-lamp biomicroscopy of the anterior eye segment, fundus examination and spectral-domain optical coherence tomography (OCT) imaging. Longitudinal analysis focused on the central retinal thickness (CRT), peripapillary retinal nerve fiber layer (RNFL) thickness, intraretinal hyperreflective foci (HRF), and retinal vessel tortuosity.

**Results:**

Over a median follow-up period of 6.5 years, BCVA remained stable in all patients. Serum lyso-globotriaosylceramide (lyso-Gb3) levels demonstrated strong correlations with intraretinal HRF and retinal vessel tortuosity but no significant change during the observation period was observed. Longitudinal assessment showed no significant change of HRF, retinal vessel tortuosity or CRT over time, whereas a significant reduction of RNFL thickness was observed, although these values remained within the normal range.

**Conclusion:**

Long-term treatment with ERT or PCT in FD is associated with a largely stable retinal phenotype. Retinal imaging parameters remain structurally preserved over time, with only mild RNFL thinning observed, suggesting limited progressive retinal neurodegeneration.

## Introduction

Fabry disease (FD) is a rare X-linked lysosomal storage disorder caused by pathogenic variants in the *GLA* gene, resulting in a deficiency of the soluble lysosomal enzyme α-galactosidase A [1, 2]. This enzymatic deficiency results in tissue accumulation of globotriaosylceramide (Gb3), in the vascular endothelium, kidneys, heart, and nervous system. Subsequently, this may cause severe and potentially life-threatening complications, such as kidney failure, heart failure, and cerebrovascular events [3–7]. The phenotype is highly variable, ranging from late-onset oligosymptomatic patients to severe and progressive multi-organ involvement [1, 5, 7]. The disease onset commonly occurs in childhood or adolescence, with often misinterpreted symptoms as gastrointestinal symptoms, acroparesthesia and angiokeratomas, leading to diagnostic delay. Women and girls can also be affected despite X-linked inheritance, but their symptoms are often milder and appear later in life.

The ocular phenotype of FD includes cornea verticillata, conjunctival and retinal vascular tortuosity, posterior spoke-like cataract, and intraretinal hyperreflective foci (HRF) [8–16]. These features may facilitate diagnosis but may also represent biomarkers of systemic involvement and disease progression [13, 17]. Intraretinal HRF, observed within the inner retinal layers (ganglion cell layer, inner plexiform layer, inner nuclear layer, outer plexiform layer), are thought to reflect intraretinal deposits and capillary dysfunction and show a strong correlation with lyso-Gb3 levels, a key biomarker of disease burden and progression [11]. It has been hypothesized that HRF represent local lyso-Gb3 accumulation, lipid depositions or perivascular inflammatory activity/markers of microvascular remodeling [15, 16, 18]. Retinal vessel tortuosity has been shown to correlate with systemic involvement and may be evaluated and graded using AI-based image analyses [19, 20]. 

Disease-specific therapies may modify disease progression and improve patient-reported as well as organ-specific outcomes in FD [21]. Enzyme replacement therapies (ERT), replace deficient α-galactosidase A and reduce lyso-Gb3 levels, resulting in stabilization of renal function and improvements in cardiac manifestation and reduced pain, however, such therapies have limitations such as infusion burden and immune-mediated reactions [22–24]. Pharmacological chaperone therapy (PCT) with migalastat represents an alternative oral therapeutic approach for patients with amenable mutations, stabilizing endogenous α-galactosidase A, enhancing enzyme activity, and offering a less invasive treatment option [25–28]. For all therapeutic approaches, treatment selection is guided by the patient’s genetic background, clinical phenotype, treatment tolerance, and patient preferences [21]. 

Even though ERT and PCT are central for the treatment of FD, their effects on the ocular phenotype remain incompletely understood [29]. This study characterized the retinal phenotype under continuous systemic treatment longitudinally, to better understand the ocular involvement and its potential role as a non-invasive biomarker.

## Materials and methods

This retrospective study was performed at the Department of Ophthalmology at the University Medical Center Hamburg-Eppendorf, Germany and was in adherence to the Declaration of Helsinki. This study was approved by the medical ethics committee of the Ärztekammer Hamburg (PV5470), Germany. Patients and/or parents’ consent was obtained.

### Ocular examination

All subjects underwent a comprehensive ocular examination including best-corrected visual acuity (BCVA) testing, tonometry, slit-lamp biomicroscopy assessing the anterior segment with a focus on cornea verticillata, conjunctival vessel tortuosity, lens opacification, and fundoscopy.

### Retinal imaging and image analysis

Retinal imaging included fundus photography and optical coherence tomography (OCT, Spectralis, Heidelberg Engineering, Heidelberg, Germany). The central retinal thickness (CRT) was evaluated using a 20° x 20° macular OCT-scan grid and data were examined based on the subfields as defined by the Early Treatment Diabetic Retinopathy Study. The thickness of the peripapillary retinal nerve fiber layer (RNFL) was assessed based on OCT imaging and was performed around the optic nerve in a circular band approximately 3.4 mm in diameter. Images were processed and analyzed using ImageJ software (U.S. National Institutes of Health, Bethesda, Maryland, USA). Averaged CRT and RNFL measurements from both eyes of each subject were calculated for statistical correlation analysis.

### HRF

**Fig. 1:**
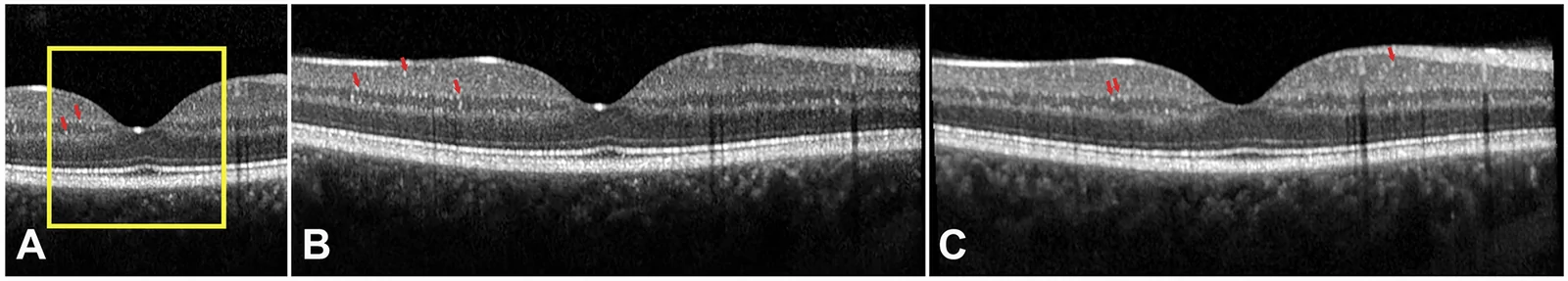
OCT imaging of right and left eye of patient 4. (**A**) The parafoveal region, magnified at 300%, reveals numerous HRFs (red arrows) within the inner retinal layers. Quantification was performed by measuring 1000 μm from the center towards both the nasal and temporal sides, creating a total width of 2000 μm around the fovea. (**B**) Hyperreflective changes at initial presentation and (**C**) six years later are displayed

Quantification of HRF was performed as previously reported [11]. In short, the temporal and nasal parafoveal regions (0–1,000 μm) were outlined in a foveal cross-section using ImageJ. These regions were quantified using the counting tool at 300% magnification, as shown in Fig. 1A. HRF, identified by their similar reflectivity to the retinal pigment epithelium were counted. All potential HRFs with shadows were excluded to prevent misidentification between retinal arterioles and venules. A clinical grading of HRF was performed, rating each case as absent (0), mild [1], moderate [2], or severe [3], based on the visual assessment of the OCT images by two independent ophthalmologists (Fig. 1B and C).

### Retinal vessel tortuosity

**Fig. 2:**
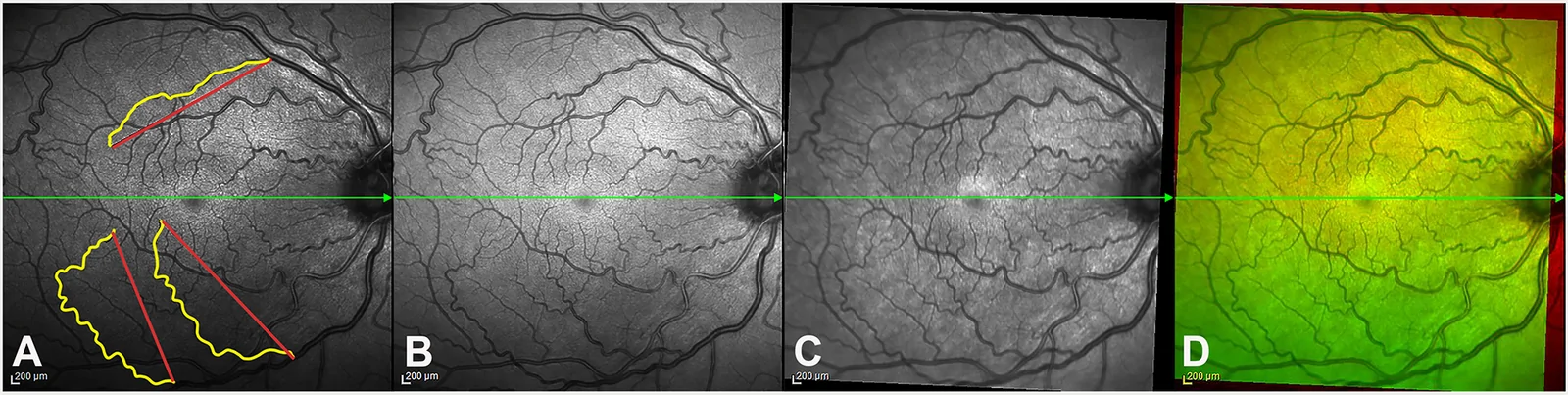
cSLO images of the right eye of patient 9. (**A**) Objective score of retinal vessel tortuosity was analyzed by measuring the true length (a,yellow) and the end-to-end length (b,red) of three retinal vessels and dividing a/b. (**B**) Baseline fundus photograph of the right eye of patient 9. (**C**) Follow-up fundus photograph of the same eye acquired five years later under continuous ERT. (**D**) Overlay of both images, revealing an almost unchanged retinal vascular pattern over time

Retinal vessel tortuosity was analyzed based on confocal scanning laser ophthalmoscopy (cSLO) images captured during the OCT imaging. Each image was reviewed to identify the three most tortuous vessels. These vessels were used to calculate the vessel tortuosity score by measuring the length (a) and the end-to-end length (b) of each vessel, with the ratio a/b providing a quantified score, as shown in Fig. 2A. Images were graded by two ophthalmologists, the vessel tortuosity was graded as absent (0), mild [1], moderate [2], or severe [3].

### Systemic parameters

The serum biomarker lyso-Gb3, which correlates with the severity of FD [30], was obtained from all patients.

### Statistical analysis

Statistical analyses were performed using IBM SPSS Statistics software (version 29.0.1.0 (171), IBM Corporation, Armonk, New York). The significance level was set at *p* < 0.05 for all statistical tests. For the comparison of retinal parameters between initial and follow-up examination, the Wilcoxon-Mann-Whitney U-test was used for non-normally distributed data and the dependent T-test for normally distributed data. Spearman rank order test was used for correlation analyses.

## Results

Baseline assessments were available for 56 eyes (28 patients) with a median age of 48 years (range: 10–65 years; standard deviation (SD): 14.2 years). Due to loss of follow-up (*n* = 4) or discontinuation of systemic therapy (*n* = 3), 42 eyes (21 patients, 48% females) were available for longitudinal assessment. Out of those patients, 71% had classic Fabry variants, 29% late-onset variants. Classic variants were observed in 82% (9 of 11) of males and 60% of females (6 of 10). The median follow-up period was 6.5 years (range: 5–8 years). All patients were under continuous ERT or PCT during this period. The demographic data is presented in Table 1.

**Table 1:** Demographic and clinical characteristics of the study population

ID	Sex	age at initial examination (years)	age at last examination (years)	Treatment duration (years)	ophthalmic observation period (years)	clinical phenotyp	Therapy
1	f	31	38	8,8	7,2	classic	PCT
2	f	56	63	16,5	7,4	classic	ERT
3	m	26	31	16,6	5,3	classic	ERT
4	m	27	33	15,9	6,5	classic	ERT
5	f	52	58	2,8	5,9	late-onset	PCT
6	m	10	17	8,7	7,4	late-onset	ERT
7	f	40	46	8,7	6,4	late-onset	ERT
8	m	39	44	13,3	6,5	classic	ERT
9	m	33	40	12,9	7,2	classic	ERT
10	f	56	62	12,9	6,4	classic	ERT
11	m	59	63	8,4	6,0	classic	ERT
12	f	25	31	8,7	6,5	late-onset	ERT
13	f	49	57	8,8	8,5	late-onset	ERT
14	m	65	71	7,5	6,7	late-onset	ERT and PCT
15	f	48	54	9,8	6,2	classic	ERT
16	m	56	62	17,9	6,2	classic	ERT
17	f	52	58	11,2	5,6	classic	ERT
18	m	51	57	9,6	6,3	classic	PCT
19	f	59	65	13,5	5,5	classic	ERT
20	m	37	44	17,9	7,5	classic	ERT
21	m	40	47	17,0	6,7	classic	ERT

### Visual acuity

Median visual acuity of all 21 patients was 20/20 (range: 20/50 to 20/20 Snellen equivalent). No changes of the visual acuity were observed during the longitudinal assessment (median BCVA at last follow up: 20/20, *p* = 0.9).

### HRF

Measurement of quantitative and subjective HRF remained almost stable over the follow-up period. Mean quantitative HRF values showed no significant change from baseline to last follow-up (78.9 vs. 77.7; *p* = 0.7); subjective HRF scores remained overall unchanged (1.9 vs. 1.9; *p* = 1.0). Male patients consistently exhibited more HRF than females, with a quantitative HRF value of 95.8 vs. 60.3 at baseline and 93.2 vs. 60.7 at last follow-up. Similarly, subjective HRF scores were higher in males than females at baseline (2.6 vs. 1.3) and last follow-up (2.5 vs. 1.4). No significant longitudinal changes were observed within either sex subgroup for quantitative or subjective HRF measurements (males: quantitative *p* = 0.63, subjective *p* = 0.51; females: quantitative *p* = 0.83, subjective *p* = 0.75). These findings were consistent irrespective of treatment modality, including ERT, PCT, or combination therapy (Table 2).

**Table 2:** Baseline and last follow-up values of investigated key retinal parameters

	initial examination			follow up examination			Paired *N* for *p*	*p*-value
	*N*	Min/Max	DIST. (mean)	N2	Min/Max2	DIST. (mean)2		
Central retinal thickness (CRT)	20	241.5/305.5	277,2	21	231.5/326.5	278,4	20	0,40
Retinal nerve fiber layer thickness (RNFL)	21	83/117	96,5	20	76/112	93,5	20	0,01
BCVA / Visus	21	0.43/1	0,9	21	0.43/1	0,9	21	0,96
Objective calculated vessel tortuosity score	21	1.09/1.6	1,2	21	1.09/1.6	1,2	21	1,00
Subjective obtained vessel tortuosity score	21	0/3	1,4	21	0/3	1,4	21	1,00
Quantitative score of hyperreflective intraretinal deposits	21	33/141	78,9	21	36/116	77,7	21	0,68
Subjective obtained score of hyperreflective intraretinal deposits	21	0.5/3	1,9	21	0.5/3	1,9	21	1,00

### Vascular tortuosity

The average objective and subjective tortuosity score at baseline visit were 1.2 and 1.4, respectively. Both scores remained stable at last follow up visit. At baseline and at last follow-up scores were higher in males (1.3 and 2.1) compared to the females (1.1 and 0.8). Longitudinal assessment of retinal vascular tortuosity revealed overall no changes over the observation period (*p* = 1.0) (Table 2; Fig. 2B, C and D).

### CRT and RNFL

**Fig. 3:**
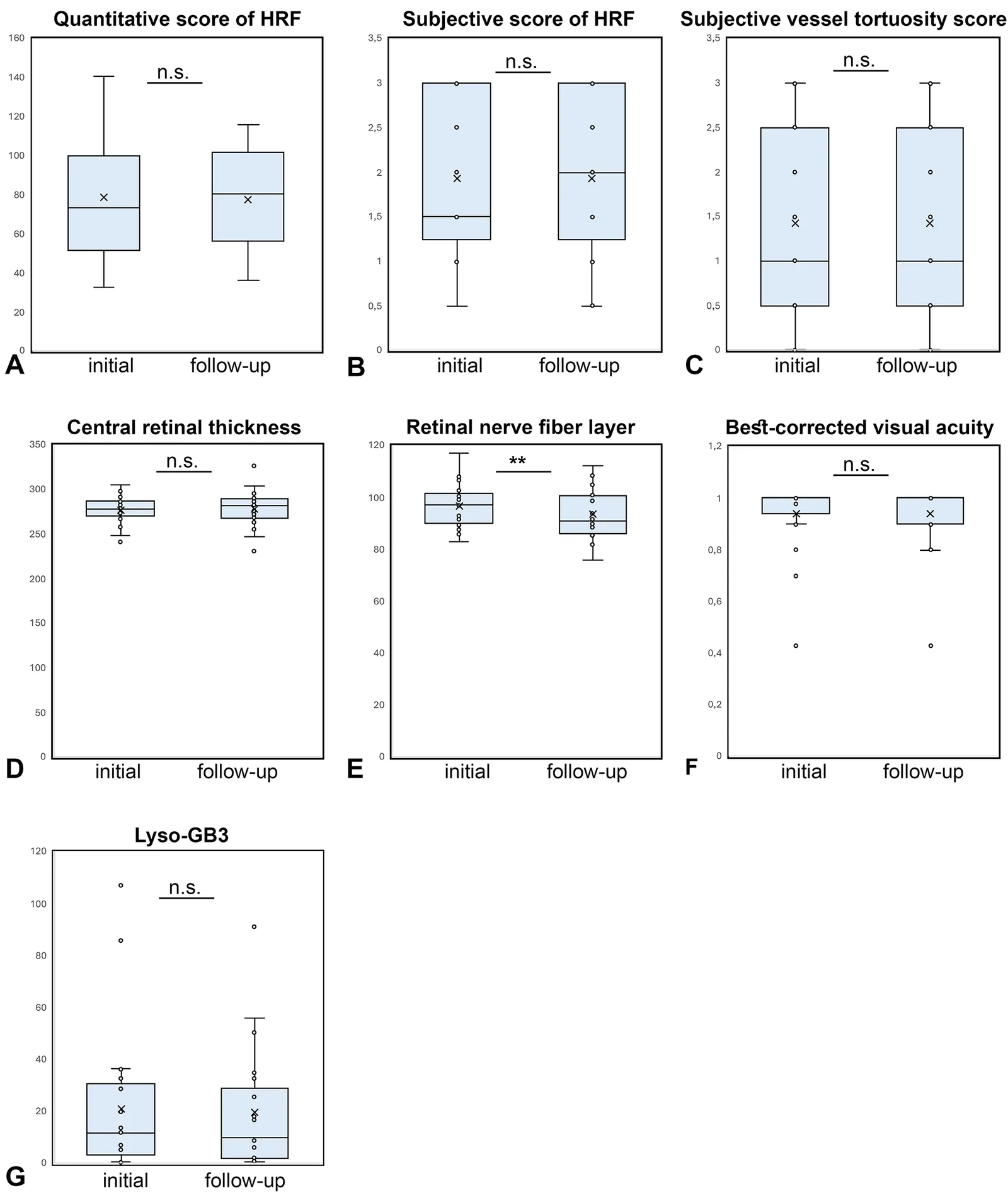
Comparative analysis of examination results at initial presentation and after 6.5 Years. Figure (**a**) compares counts of hyperreflective foci (*p* = 0.68); (**b**) compares hyperreflective foci using a subjective scoring system (*p* = 1.00); (**c**) compares vessel tortuosity with a subjective score (*p* = 1.00); (**d**) compares central retinal thickness (*p* = 0.39); (**e**) compares retinal nerve fiber layer (RNFL) thickness (*p* = 0.01); (**f**) compares best corrected visual acuity (*p* = 0.94); (**g**) compares Lyso-Gb3 levels (*p* = 0.25)

Longitudinal analysis of CRT (*p* = 0.4) demonstrated no significant changes during the follow-up period without any differences between sexes. CRT was within the normal age-range for all individuals. However, a reduction in RNFL thickness was observed over time (*p* = 0.01) although values remained within the normal age-range throughout (Fig. 3). The mean RNFL loss was 2.9 μm for males (SD: 2.3 μm) and 2.8 μm for females (SD: 5.9 μm) (Table 2).

### Lyso-Gb3 levels

A strong correlation was observed between lyso-Gb3 levels, an established biomarker for FD severity, and vascular tortuosity (*r* = 0,89; *p* < 0,001) as well as HRF (*r* = 0,74; *p* < 0,001). Over the observation period, lyso-Gb3 levels showed a mean decrease of 1.57 nmol/L (SD: 15.4 nmol/L; *p* = 0.25), which may resemble variability in patients undergoing continuous therapy. Comparative analysis of ophthalmologic and systemic parameters between initial visit and last follow-up is shown in Fig. 3.

### Subgroup analysis

Subgroup analysis comparing male and female patients with the classic or late-onset phenotype yielded consistent results, which demonstrated no significant differences over time across all subgroups in quantitative and subjective scores of HRF, objective and subjective vessel tortuosity scores, visual acuity, CRT and RNFL. Due to the low number of included patients (*n* = 2), males with the late-onset phenotype were not analyzed separately.

## Discussion

This study describes the longitudinal course of retinal findings in patients with FD receiving continuous ERT or PCT. Hyperreflective foci and retinal vessel tortuosity, which are characteristic retinal features of FD, remained stable over time, together with other structural retinal parameters. These findings provide longitudinal retinal imaging-based evidence of overall stable retinal morphology in treated FD patients despite continuous therapy.

Systemic treatment of FD with ERT or PCT has been shown to reduce lyso-Gb3 levels and slow the progression of renal and cardiac involvement, particularly when initiated before irreversible organ damage has occurred [19, 25, 28, 31–42]. However, established cardiac fibrosis, hypertrophy, and vascular remodeling are often only partially reversible despite sustained biochemical response [20, 34, 35, 40–42]. Histology shows that ERT substantially clears Gb3 from many cells, but podocytes and cardiomyocytes are difficult to fully clear, especially in advanced disease [32–34]. Our findings suggest that retinal manifestations may follow a similar pattern, remaining structurally stable under long-term therapy without evidence of regression.

Previous longitudinal data on ocular manifestations in FD are limited. A prospective observational study analyzing mainly anterior segment findings over five years reported progression of corneal haze, cataract formation, and microaneurysms despite systemic therapy in the majority of patients, whereas retinal vessel tortuosity remained largely stable [29]. In agreement with our findings, the authors observed no significant longitudinal progression in the severity of retinal vascular changes, supporting the concept that retinal manifestations in FD may represent chronic structural alterations rather than reversible treatment-responsive findings.

In previous reports strong correlation between HRF and plasma lyso-Gb3 levels have been shown, suggesting that HRF may reflect systemic glycosphingolipid accumulation and disease severity [11, 43]. Also, higher HRF numbers were found in patients with greater retinal vessel tortuosity and worse renal function [44], underlining that they might serve as a surrogate marker of systemic disease severity. It has been hypothesized that HRF represent Gb3 accumulation in retinal vessel walls or associated cells within the macular capillary plexuses [11, 15, 18]. Until now, no longitudinal FD data on the retinal phenotype is available and it has not been understood whether HRF regress or progress under ERT or PCT.

In the reported cohort, both quantitative and subjective HRF remained stable over time despite continuous systemic therapy. This could indicate that HRF are structural alterations and rather not dynamic treatment-responsive changes, although a delayed retinal “washout” under long-term therapy cannot be excluded with the here reported data. The association with laso-Gb3 as an established systemic biomarker suggests a potential role of HRF as ocular biomarkers of disease involvement. However, larger longitudinal studies are required to determine their suitability as imaging-based outcome parameters for disease monitoring and therapeutic response [11, 15, 18, 37]. 

Retinal vessel tortuosity likewise remained stable throughout the reported follow-up. Previous studies demonstrated correlations between retinal vessel tortuosity, lyso-Gb3 levels, Mainz Severity Score Index values, and cerebral small vessel disease, supporting its role as a marker of systemic involvement [9, 17, 20, 45]. Consistent with prior observations, retinal vessel tortuosity showed neither significant progression nor reduction over time [29, 46]. This may suggest that retinal vessel tortuosity rather reflects chronic vascular remodeling secondary to glycosphingolipid storage than a dynamically reversible process [9, 17, 20, 45]. Although the underlying pathophysiology remains incompletely understood, retinal vessel tortuosity may represent a structural marker of cumulative disease burden. Larger longitudinal studies are needed to determine its value as a monitoring parameter during systemic therapy.

CRT remained unchanged throughout follow-up, consistent with our previous cross-sectional findings showing no significant differences compared with healthy controls [11]. RNFL thickness demonstrated a mild reduction over time within the range of age-related physiological variation [47]. The progression may resemble the physiological reduction of the RNFL that occurs with ageing. Another hypothesis could be that RNFL reduction is associated with the persistence of HRF, given that the foci are located in the inner retinal layers. Visual acuity remained stable in nearly all patients, consistent with previous reports indicating that ocular manifestations in FD rarely impair visual function [11, 15, 19]. Overall, these findings suggest preserved retinal structure and visual function under continuous systemic therapy.

The potential value of non-invasive retinal imaging is that the ocular and non-ocular involvement can be determined. OCT-derived parameters such as HRF and retinal vessel tortuosity may provide accessible biomarkers reflecting systemic involvement and cumulative disease burden. Given the multisystemic nature of FD, retinal imaging may complement established systemic biomarkers and contribute to longitudinal disease monitoring. Automated image analysis approaches may improve the reproducibility, objectivity, and clinical applicability of these retinal parameters for longitudinal monitoring and therapeutic assessment [16, 17]. Future studies may integrate retinal imaging findings with systemic clinical outcomes and biochemical markers to further define their role in disease stratification and treatment monitoring.

Limitations of this study include the limited cohort size and the small patient numbers available for subgroup analyses, particularly among late-onset male patients. In addition, the absence of an untreated control group limits conclusions regarding treatment-specific effects on retinal morphology. The heterogeneous genetic and clinical characteristics of the cohort may also have influenced the observed findings. Furthermore, the observational design and relatively limited sample size restrict the generalizability of the results, emphasizing the need for larger multicenter longitudinal studies.

In conclusion, retinal manifestations in FD remained largely stable during long-term enzyme replacement or PCT. Neither significant progression nor reversal of retinal alterations was observed over time. The correlation of HRF and retinal vessel tortuosity with systemic disease markers support their potential role as non-invasive biomarkers of disease involvement and progression in FD.

## Data Availability

No datasets were generated or analysed during the current study.

## References

[1] El-Abassi R, Singhal D, England JD. Fabry’s disease. J Neurol Sci. 2014;344(1–2):5–19. 10.1016/j.jns.2014.06.029 25106696

[2] Saeed S, Imazio M. Fabry disease: Definition, Incidence, Clinical presentations and Treatment - Focus on cardiac involvement. Pak J Med Sci. 2022;38(8):2337–44. 10.12669/pjms.38.8.7063 PMC9676584 36415271

[3] Fellgiebel A, Muller MJ, Ginsberg L. CNS manifestations of Fabry’s disease. Lancet Neurol. 2006;5(9):791–5. 10.1016/S1474-4422(06)70548-8 16914407

[4] Linhart A, Kampmann C, Zamorano JL, Sunder-Plassmann G, Beck M, Mehta A, et al. Cardiac manifestations of Anderson-Fabry disease: results from the international Fabry outcome survey. Eur Heart J. 2007;28(10):1228–35. 10.1093/eurheartj/ehm153 17483538

[5] Hoffmann B, Mayatepek E. Fabry disease-often seen, rarely diagnosed. Dtsch Arztebl Int. 2009;106(26):440–7. 10.3238/arztebl.2009.0440 PMC2704393 19623315

[6] Choi JH, Lee BH, Heo SH, Kim GH, Kim YM, Kim DS, et al. Clinical characteristics and mutation spectrum of GLA in Korean patients with Fabry disease by a nationwide survey: Underdiagnosis of late-onset phenotype. Med (Baltim). 2017;96(29):e7387. 10.1097/MD.0000000000007387 PMC5521888 28723748

[7] Germain DP. Fabry disease. Orphanet J Rare Dis. 2010;5:30. 10.1186/1750-1172-5-30 PMC3009617 21092187

[8] Orssaud C, Dufier J, Germain D. Ocular manifestations in Fabry disease: a survey of 32 hemizygous male patients. Ophthalmic Genet. 2003;24(3):129–39. 10.1076/opge.24.3.129.15609 12868031

[9] Hangartner K, Bajka A, Wiest MRJ, Sidhu S, Toro MD, Maloca PM, Zweifel SA. Assessment of Retinal Vessel Tortuosity Index in Patients with Fabry Disease Using Optical Coherence Tomography Angiography (OCTA). Diagnostics (Basel). 2023;13:15. 10.3390/diagnostics13152496 PMC10417007 37568859

[10] van der Tol L, Sminia ML, Hollak CE, Biegstraaten M. Cornea verticillata supports a diagnosis of Fabry disease in non-classical phenotypes: results from the Dutch cohort and a systematic review. Br J Ophthalmol. 2016;100(1):3–8. 10.1136/bjophthalmol-2014-306433 25677671

[11] Atiskova Y, Rassuli R, Koehn AF, Golsari A, Wagenfeld L, du Moulin M, et al. Retinal hyperreflective foci in Fabry disease. Orphanet J Rare Dis. 2019;14(1):296. 10.1186/s13023-019-1267-2 PMC6933914 31878969

[12] Mauhin W, Benveniste O, Amelin D, Montagner C, Lamari F, Caillaud C, et al. Cornea verticillata and acroparesthesia efficiently discriminate clusters of severity in Fabry disease. PLoS ONE. 2020;15(5):e0233460. 10.1371/journal.pone.0233460 PMC7244174 32442237

[13] Gambini G, Scartozzi L, Giannuzzi F, Carla MM, Boselli F, Caporossi T et al. Ophthalmic manifestations in fabry disease: updated review. J Pers Med. 2023;13(6). 10.3390/jpm13060904 PMC10305323 37373893

[14] Pitz S, Kalkum G, Arash L, Karabul N, Sodi A, Larroque S, et al. Ocular signs correlate well with disease severity and genotype in Fabry disease. PLoS ONE. 2015;10(3):e0120814. 10.1371/journal.pone.0120814 PMC4363518 25781336

[15] Wu Y, Zhang W, Yao X, Song W, Zhao Y, Yuan Y, Zhang W. Investigation of ocular involvement in patients with Fabry disease. Ann Med. 2023;55(1):2226909. 10.1080/07853890.2023.2226909 PMC10291927 37354009

[16] Yanik O, Ciki K, Ozmert E, Sivri S. Vascular and structural analyses of retinal and choroidal alterations in Fabry disease: the effect of hyperreflective foci and retinal vascular tortuosity. Ophthalmic Genet. 2022;43(3):344–53. 10.1080/13816810.2022.2025607 35038962

[17] Sodi A, Guarducci M, Vauthier L, Ioannidis AS, Pitz S, Abbruzzese G, et al. Computer assisted evaluation of retinal vessels tortuosity in Fabry disease. Acta Ophthalmol. 2013;91(2):e113–9. 10.1111/j.1755-3768.2012.02576.x 23164241

[18] Moon SS, Ovens CA, Reid G, Fraser CL. SD-OCT Imaging of Macular Changes in Fabry Disease: A Case Report. Neuroophthalmology. 2024;48(3):198–203. 10.1080/01658107.2024.2301921 PMC11095570 38756337

[19] Sodi A, Ioannidis AS, Mehta A, Davey C, Beck M, Pitz S. Ocular manifestations of Fabry’s disease: data from the Fabry Outcome Survey. Br J Ophthalmol. 2007;91(2):210–4. 10.1136/bjo.2006.100602 PMC1857640 16973664

[20] Atiskova Y, Wildner J, Spitzer MS, Aries C, Muschol N, Dulz S. Retinal vessel tortuosity as a prognostic marker for disease severity in Fabry disease. Orphanet J Rare Dis. 2021;16(1):485. 10.1186/s13023-021-02080-0 PMC8605526 34801073

[21] Mignani R, Biagini E, Cianci V, Pieruzzi F, Pisani A, Tuttolomondo A, Pieroni M. Effects of Current Therapies on Disease Progression in Fabry Disease: A Narrative Review for Better Patient Management in Clinical Practice. Adv Ther. 2025;42(2):597–635. 10.1007/s12325-024-03041-2 PMC11787255 39636569

[22] Mehta A, Beck M, Eyskens F, Feliciani C, Kantola I, Ramaswami U, et al. Fabry disease: a review of current management strategies. QJM. 2010;103(9):641–59. 10.1093/qjmed/hcq117 20660166

[23] Tsuboi K, Yamamoto H. Efficacy and safety of enzyme-replacement-therapy with agalsidase alfa in 36 treatment-naive Fabry disease patients. BMC Pharmacol Toxicol. 2017;18(1):43. 10.1186/s40360-017-0152-7 PMC5463328 28592315

[24] Tondel C, Thurberg BL, DasMahapatra P, Lyn N, Maski M, Batista JL, et al. Clinical relevance of globotriaosylceramide accumulation in Fabry disease and the effect of agalsidase beta in affected tissues. Mol Genet Metab. 2022;137(4):328–41. 10.1016/j.ymgme.2022.10.005 36334424

[25] Weidemann F, Jovanovic A, Herrmann K, Vardarli I. Chaperone Therapy in fabry disease. Int J Mol Sci. 2022;23(3). 10.3390/ijms23031887 PMC8836454 35163813

[26] Yam GH, Bosshard N, Zuber C, Steinmann B, Roth J. Pharmacological chaperone corrects lysosomal storage in Fabry disease caused by trafficking-incompetent variants. Am J Physiol Cell Physiol. 2006;290(4):C1076–82. 10.1152/ajpcell.00426.2005 16531566

[27] Germain DP, Hughes DA, Nicholls K, Bichet DG, Giugliani R, Wilcox WR, et al. Treatment of Fabry’s Disease with the Pharmacologic Chaperone Migalastat. N Engl J Med. 2016;375(6):545–55. 10.1056/NEJMoa1510198 27509102

[28] Hughes DA, Nicholls K, Shankar SP, Sunder-Plassmann G, Koeller D, Nedd K, et al. Oral pharmacological chaperone migalastat compared with enzyme replacement therapy in Fabry disease: 18-month results from the randomised phase III ATTRACT study. J Med Genet. 2017;54(4):288–96. 10.1136/jmedgenet-2016-104178 PMC5502308 27834756

[29] Michaud L. Longitudinal study on ocular manifestations in a cohort of patients with Fabry disease. PLoS ONE. 2019;14(6):e0213329. 10.1371/journal.pone.0213329 PMC6597042 31246960

[30] Nowak A, Mechtler TP, Hornemann T, Gawinecka J, Theswet E, Hilz MJ, Kasper DC. Genotype, phenotype and disease severity reflected by serum LysoGb3 levels in patients with Fabry disease. Mol Genet Metab. 2018;123(2):148–53. 10.1016/j.ymgme.2017.07.002 28728877

[31] Anderson LJ, Wyatt KM, Henley W, Nikolaou V, Waldek S, Hughes DA, et al. Long-term effectiveness of enzyme replacement therapy in Fabry disease: results from the NCS-LSD cohort study. J Inherit Metab Dis. 2014;37(6):969–78. 10.1007/s10545-014-9717-4 24831586

[32] Bichet DG, Aerts JM, Auray-Blais C, Maruyama H, Mehta AB, Skuban N, et al. Assessment of plasma lyso-Gb(3) for clinical monitoring of treatment response in migalastat-treated patients with Fabry disease. Genet Med. 2021;23(1):192–201. 10.1038/s41436-020-00968-z PMC7790748 32994552

[33] Braun F, Abed A, Sellung D, Rogg M, Woidy M, Eikrem O et al. Accumulation of alpha-synuclein mediates podocyte injury in fabry nephropathy. J Clin Invest. 2023;133(11). 10.1172/JCI157782 PMC10232004 37014703

[34] Frustaci A, Verardo R, Galea N, Alfarano M, Magnocavallo M, Marchitelli L, et al. Long-Term Clinical-Pathologic Results of Enzyme Replacement Therapy in Prehypertrophic Fabry Disease Cardiomyopathy. J Am Heart Assoc. 2024;13(8):e032734. 10.1161/JAHA.123.032734 PMC11262509 38563373

[35] Kim JH, Lee BH, Hyang Cho J, Kang E, Choi JH, Kim GH, Yoo HW. Long-term enzyme replacement therapy for Fabry disease: efficacy and unmet needs in cardiac and renal outcomes. J Hum Genet. 2016;61(11):923–9. 10.1038/jhg.2016.78 27334365

[36] Laffer B, Lenders M, Ehlers-Jeske E, Heidenreich K, Brand E, Kohl J. Complement activation and cellular inflammation in Fabry disease patients despite enzyme replacement therapy. Front Immunol. 2024;15:1307558. 10.3389/fimmu.2024.1307558 PMC10830671 38304433

[37] Lenders M, Nowak A, Cybulla M, Kaufeld J, Kohn AF, Muschol NM, et al. Impact of enzyme replacement therapy on clinical manifestations in females with Fabry disease. Orphanet J Rare Dis. 2024;19(1):490. 10.1186/s13023-024-03503-4 PMC11673826 39731156

[38] Riccio E, Zanfardino M, Ferreri L, Santoro C, Cocozza S, Capuano I, et al. Switch from enzyme replacement therapy to oral chaperone migalastat for treating fabry disease: real-life data. Eur J Hum Genet. 2020;28(12):1662–8. 10.1038/s41431-020-0677-x PMC7784987 32647377

[39] Rombach SM, Smid BE, Bouwman MG, Linthorst GE, Dijkgraaf MG, Hollak CE. Long term enzyme replacement therapy for Fabry disease: effectiveness on kidney, heart and brain. Orphanet J Rare Dis. 2013;8:47. 10.1186/1750-1172-8-47 PMC3626869 23531228

[40] Tsujiuchi M, Ebato M, Maezawa H, Mizukami T, Nogi A, Ikeda N, et al. Long-Term Effects of Enzyme Replacement Therapy for Anderson-Fabry Disease. Int Heart J. 2019;60(1):208–14. 10.1536/ihj.17-688 30464119

[41] Weidemann F, Niemann M, Stork S, Breunig F, Beer M, Sommer C, et al. Long-term outcome of enzyme-replacement therapy in advanced Fabry disease: evidence for disease progression towards serious complications. J Intern Med. 2013;274(4):331–41. 10.1111/joim.12077 PMC4282332 23586858

[42] Rombach SM, Smid BE, Linthorst GE, Dijkgraaf MG, Hollak CE. Natural course of Fabry disease and the effectiveness of enzyme replacement therapy: a systematic review and meta-analysis: effectiveness of ERT in different disease stages. J Inherit Metab Dis. 2014;37(3):341–52. 10.1007/s10545-014-9677-8 24492980

[43] Mat Nor MN, Green CR, Squirrell D, Acosta ML. Retinal Hyperreflective Foci Are Biomarkers of Ocular Disease: A Scoping Review With Evidence From Humans and Insights From Animal Models. J Ophthalmol. 2025;2025:9573587. 10.1155/joph/9573587 PMC12140829 40474962

[44] Bacherini D, Mercuri S, Sodi A, Gambini G, Rizzo C, Rizzo S, et al. Inner retinal hyper-reflective foci number correlates with flood-illumination adaptive optics ophthalmoscopy grading and systemic organ involvement in patients with Fabry disease. Eye (Lond). 2025;39(8):1578–83. 10.1038/s41433-025-03721-5 PMC12089511 39979614

[45] Li Y, Zhou X, Li J, Zhao Y, Yuan Y, Yang B, et al. Deep learning assisted retinal microvasculature assessment and cerebral small vessel disease in Fabry disease. Orphanet J Rare Dis. 2025;20(1):158. 10.1186/s13023-025-03627-1 PMC11969690 40181436

[46] Fledelius HC, Sandfeld L, Rasmussen AK, Madsen CV, Feldt-Rasmussen U. Ophthalmic experience over 10 years in an observational nationwide Danish cohort of Fabry patients with access to enzyme replacement. Acta Ophthalmol. 2015;93(3):258–64. 10.1111/aos.12588 25487570

[47] Parikh RS, Parikh SR, Sekhar GC, Prabakaran S, Babu JG, Thomas R. Normal age-related decay of retinal nerve fiber layer thickness. Ophthalmology. 2007;114(5):921–6. 10.1016/j.ophtha.2007.01.023 17467529

